# Health of Aging Families: Comparing Compound and Noncompound Caregivers

**DOI:** 10.1093/geroni/igab046.397

**Published:** 2021-12-17

**Authors:** Fei Wang, Christina Marsack-Topolewski, Rosanne DiZazzo-Miller, Preethy Samuel

**Affiliations:** 1 Case Western Reserve University, Cleveland Heights, Ohio, United States; 2 Eastern Michigan University, Ypsilanti, Michigan, United States; 3 Wayne State University, Detroit, Michigan, United States

## Abstract

Providing care to a family member with disabilities takes a toll on the caregiver and the whole family's health. Among aging caregivers, compound caregiving (i.e., caring for additional family members) has become an increasingly common scenario. However, few research studies have focused on compound caregivers. Extant literature describes individual-level outcomes, with sparse knowledge on family-level outcomes. The present study examines the differences in the individual and family health of aging compound and noncompound caregivers, using the family quality of life framework. Web-based cross-sectional data collected from 112 aging caregivers (i.e., over 50 years) was analyzed using chi-square and independent sample t-tests to examine differences between caregivers. Compound caregivers had worse perceptions of personal health (t = -2.96, p = .004, d = -.61) than noncompound caregivers. In terms of family health, although all caregivers shared similar perceptions on the importance, opportunities, initiative, and stability, compound caregivers had lower attainment (t = -2.64, p = .009, d = -.44) and satisfaction (t = -3.90, p < .001, d = -.73) than noncompound caregivers. Findings have practice implications for identifying caregivers' multiple responsibilities. It is necessary to develop individual and family level programs focused on health promotion and caregiving training.

